# A case of incidental STUMP discovery in a patient with concurrent prostatic adenocarcinoma

**DOI:** 10.1093/jscr/rjab576

**Published:** 2021-12-30

**Authors:** Tessa Ladner, Troy Schultz, Jocelyn Moore, Greg Houle

**Affiliations:** Department of Medicine, University of British Columbia, Vancouver, BC, Canada; Department of Medicine, University of British Columbia, Vancouver, BC, Canada; Vernon Jubilee Hospital, Vernon, BC, Canada; Department of Medicine, University of British Columbia, Vancouver, BC, Canada; BC Cancer Kelowna, Sindi Ahluwalia Hawkins Centre for the Southern Interior, Kelowna, BC, Canada; Department of Medicine, University of British Columbia, Vancouver, BC, Canada; Vernon Jubilee Hospital, Vernon, BC, Canada

## Abstract

Stromal tumours of the prostate are exceedingly rare, often presenting in patients in their fifth decade of life. They are classified as either stromal sarcomas, or stromal tumours of uncertain malignant potential (STUMP), the latter of which is known to have diverse clinical behaviour and thus surgical excision is often warranted. We present a case of a 71-year-old male, initially worked up by his family doctor due to mild obstructive voiding symptoms. Following a more thorough urologic workup, including a prostate biopsy, he was found to have a markedly elevated prostate specific antigen and positive cores on prostate biopsy demonstrating prostatic adenocarcinoma. The decision was made to treat with retropubic radical prostatectomy and bilateral pelvic lymph node dissection. Resulting pathology showed concurrent prostatic adenocarcinoma in addition to STUMP. The patient continues to be followed by oncology as well as a sarcoma specialist due to the unique nature of his case.

## INTRODUCTION

Prostatic stromal tumours, arising from mesenchymal tissue, are exceedingly rare, accounting for <1% of prostate cancers [1]. As per the WHO classification, these are classified as either stromal sarcomas or stromal tumours of uncertain malignant potential (STUMP) [2]. STUMP is known to have diverse clinical behaviour, ranging from incidental discovery and no progression, to distant metastasis and even death [3]. To date, no radiological features have been demonstrated to predict aggression or prognosis of STUMP of the prostate, and thus, surgical excision is often warranted [4, 5]. There has been no consensus in the literature surrounding the relationship between prostate specific antigen (PSA) and this particular type of tumour [4, 6].

There remain relatively few case reports of concurrent prostatic stromal tumours and adenocarcinoma in the literature. Here, we present a case of suspicious magnetic resonance imaging (MRI) findings, suggestive of a complex hemorrhagic cyst, and prostate biopsy positive for prostatic adenocarcinoma, managed with radical prostatectomy and a resulting pathologic discovery of concurrent STUMP as well as adenocarcinoma. This patient in particular was unique due to his late age of onset for this particular type of tumour, as well as the concurrent diagnosis.

## CASE PRESENTATION

A 71-year-old Caucasian male presented with mild obstructive voiding symptoms to his general practitioner. Past medical history was significant for unmedicated hypertension, chronic obstructive pulmonary disease, gastroesophageal reflux disease and Reiter’s syndrome. Significantly, he had a 50 pack year history of cigarette smoking. At presentation, he denied any hematuria or signs of urinary tract infection. A PSA test was normal at 3.54 at this time. One year later, repeat PSA was found to be 17.8, prompting a referral to Urology. Workup after referral included a transrectal ultrasound guided (TRUS) prostate biopsy, revealing 2/10 cores positive for Gleason 3 + 4 = 7 prostatic adenocarcinoma. No evidence of perineural invasion, lymphovascular invasion or extraprostatic extension was identified. Staging was conducted with a bone scan and an abdominal and pelvic computed tomography scan, revealing no lymphadenopathy or bony metastases. Further investigation using an MRI of the prostate revealed a 104 cm [3] prostate, with a complex hemorrhagic or proteinaceous right anterolateral prostatic or possibly extraprostatic cyst, PI-RADS 3. There was no invasion of the rectum noted. A PSA collected 17 months after his original presentation was low, at 3.41.

**Figure 1:**
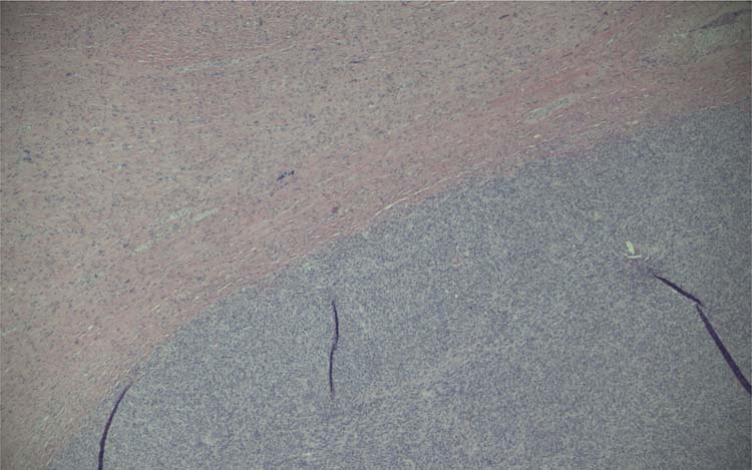
Magnification (40x) of histologic section of prostate, showing STUMP (upper left of image) and adjacent sarcoma (lower right of image).

**Figure 2:**
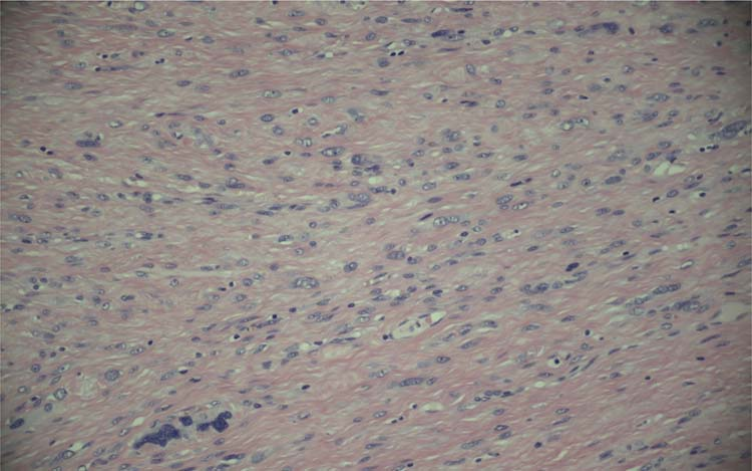
Magnification (200x) of histologic section of prostate, showing STUMP with severe cytologic atypia and no increase in mitotic activity.

**Figure 3:**
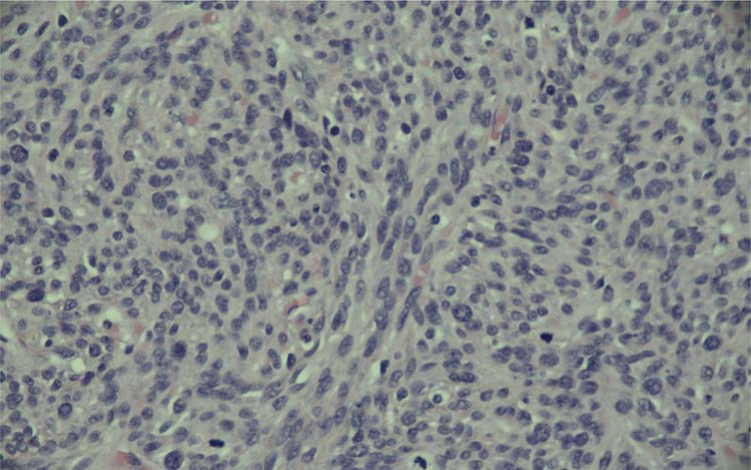
Magnification (400x) of histologic section of prostate, showing stromal sarcoma with moderate cytologic atypia and brisk mitotic activity.

Ultimately, this patient underwent a retropubic radical prostatectomy and bilateral pelvic lymph node dissection. The pathology was reviewed at BC Cancer Centre. A Gleason score 3 + 4 with tertiary 5 prostatic adenocarcinoma was found, involving ~10% of the prostate. Focal extraprostatic extension was found, with no seminal vesicle invasion. Margins were clear, and no malignancy was found in any of the 14 lymph nodes removed. This resulted in a pathologic staging of pT3aN0 (See Figs 1–3). Additionally, a stromal sarcoma was found, measuring 3.2 cm in size, located in the right anterolateral aspect of the specimen. This corresponded to the cystic lesion as seen on ultrasound and MRI. It was found to be arising from a STUMP, and although the tumour grade was favoured to be low, it was mitotically active with up to 10 mitoses per 10 high power field. Margins, again, were found to be negative.

PSA 3 months post prostatectomy was found to be normal at 0.03. This patient will be monitored by Urology moving forward and have serial PSA levels to ensure no recurrence. This case was discussed at provincial oncology rounds and a sarcoma specialist was consulted. The consensus was that no radiation therapy would be offered post-operatively, given the low post-op PSA, negative margins on pathology and lack of benefit of adjuvant radiotherapy in the current literature.

## DISCUSSION

STUMP is currently classified based on classification first proposed by Gaudin *et al.* [7], broken into four stromal patterns: cellular stromal, phyllodes, stromal predominant/myxoid or degenerative atypia pattern.

The behaviour of STUMP is unpredictable, and to date, there are no proven factors seen on imaging that indicate future progression or malignant potential with certainty. On occasion, STUMP has been known to progress to stromal sarcoma, characterized by cellular atypia, mitotic activity, necrosis and stromal hypercellularity [8]. Due to its unpredictable nature, surgical treatment is often warranted [9]. In our patient’s case, he had already elected to proceed with a radical prostatectomy due to the finding of prostatic adenocarcinoma on biopsy. Additionally, mitotic activity was found in our patient’s pathology, further confirming that radical prostatectomy was an appropriate treatment approach.

PSA values have been known to be unpredictable in cases of STUMP; some have noted elevated PSA values [7], while others have noted no correlation between PSA values and extent of disease [9]. In this case, the patient’s PSA level was within normal limits at initial presentation. One year later, it was found to be elevated, however there was suspicion of prostatitis falsely elevating his PSA level. As this patient opted to undergo radical prostatectomy, and a PSA level 17 months later had returned to normal, it is unclear whether the PSA was elevated due to his prostatic adenocarcinoma, STUMP or another process. This reiterates the need to consider multiple factors and not rely solely on PSA when investigating prostate-related urological issues.

## CONCLUSIONS

To date, although there are a number of case reports of STUMP in the literature, there are relatively few cases of concurrent prostatic adenocarcinoma and stromal sarcoma [10–12]. Our patient presents a unique case of concurrent prostatic adenocarcinoma and stromal sarcoma arising from within a STUMP. Although it was clear that a radical prostatectomy was the treatment of choice, based on both provider and patient’s perspectives’, follow-up for this unique case was more of a dilemma. There was question surrounding whether serial MRIs are warranted to evaluate for any early recurrence of the sarcomatous component of his tumour. Additionally, the potential use of radiation was discussed. Ultimately, an oncologic surgeon that specializes in sarcoma was involved in this patient’s care, and it was decided that no radiation therapy would be needed post-operatively given his low postoperative PSA, negative margins and lack of benefit of adjuvant radiotherapy in the current literature.

## References

[1] McKenney JK . Mesenchymal tumors of the prostate. Mod Pathol 2018;31:S133–42. 29297486 10.1038/modpathol.2017.155

[2] Humphrey PA, Moch H, Cubilla AL, Ulbright TM, Reuter VE. The 2016 WHO classification of tumours of the urinary system and male genital organs—part B: prostate and bladder tumours. Eur Urol 2016;70:106–19. 26996659 10.1016/j.eururo.2016.02.028

[3] Murer LM, Talmon GA. Stromal tumor of uncertain malignant potential of the prostate. Arch Pathol Lab Med 2014;138:1542–5. 25357117 10.5858/arpa.2013-0212-RS

[4] Herawi M, Epstein JI. Specialized stromal tumors of the prostate: a clinicopathologic study of 50 cases. Am J Surg Pathol 2006;30:694–704. 16723846 10.1097/00000478-200606000-00004

[5] Suzuki I, Kijima T, Owada A, Kamai T. Case of prostate stromal tumour of uncertain malignant potential where positron emission tomography with 18F-fluorodeoxyglucose was useful for surgical planning. BMJ Case Reports CP 2020;13:e235738. 10.1136/bcr-2020-235738 PMC7484856 32913066

[6] Ren FY, Lu JP, Wang J, Ye JJ, Shao CW, Wang MJ. Adult prostate sarcoma: radiological-clinical correlation. Clin Radiol 2009;64:171–7. 19103347 10.1016/j.crad.2008.07.013

[7] Gaudin PB, Rosai J, Epstein JI. Sarcomas and related proliferative lesions of specialized prostatic stroma: a clinicopathologic study of 22 cases. Am J Surg Pathol 1998;22:148–62. 9500215 10.1097/00000478-199802000-00002

[8] Nagar, M., & Epstein, J. I. (2011). Epithelial proliferations in prostatic stromal tumors of uncertain malignant potential (STUMP). Am J Surg Pathol, 35, 898–903. 21572264 10.1097/PAS.0b013e318214f2f2

[9] Sadimin ET, Epstein JI. 2016. Round cell pattern of prostatic stromal tumor of uncertain malignant potential: a subtle newly recognized variant. Hum Pathol 2015;52:68–73. 10.1016/j.humpath.2016.01.002 26980017

[10] De Berardinis E, Busetto GM, Antonini G, Giovannone R, Di Placido M, Magliocca FM, et al. Incidental prostatic stromal tumor of uncertain malignant potential (STUMP): histopathological and immunohistochemical findings. Urologia 2012;79:65–8. 22388992 10.5301/RU.2012.9099

[11] Al Tell T, Marconi L, Cathcart P, Challacombe B. Stumped by rapid symptomatic prostatic regrowth: a case report on a STUMP tumour of the prostate resected with HoLEP. Int J Surg Case Rep 2019;62:24–6. 31419733 10.1016/j.ijscr.2019.07.058 PMC6706608

[12] Roos FC, Sommer S, Hampel C, Melchior SW, Thüroff JW. Extraprostatic spindle cell stromal tumor of the prostate. Case report Urology (Ridgewood, NJ) 2008;71:1226.e13–5. 10.1016/j.urology.2007.11.098 18280548

